# Influence of Plant Phenology on Chemical Composition of *Monarda fistulosa* L. Organs and their Bioactive Properties

**DOI:** 10.1007/s11130-024-01238-y

**Published:** 2024-09-26

**Authors:** Łukasz Gontar, Anna Geszprych, Monika Sitarek-Andrzejczyk, Ewa Osińska

**Affiliations:** 1 Department of Vegetable and Medicinal Plants, Institute of Horticulture Sciences, Warsaw University of Life Sciences, 159 Nowoursynowska Street, Warsaw, 02-776 Poland; 2 Research and Innovation Centre Pro-Akademia, 9/11 Innowacyjna Street, Konstantynów Łódzki, 95-050 Poland

**Keywords:** Plant development, Plant part, Essential oil, Antibacterial and antifungal action, Phenolics, Antioxidant activity

## Abstract

*Monarda fistulosa* L. above-ground organs, collected at three phases of plant phenology, were investigated as potential raw materials for application in the food industry. They were evaluated regarding essential oil (EO) content, composition, and antimicrobial activity, as well as characteristics of phenolic fractions and antioxidant properties, which may determine health benefits and potential use in food preservation. The dominant constituent of leaf EO was carvacrol. In the inflorescence EO carvacrol content was especially high at the full flowering phase (45.12%), while during the fruit setting phase its content was lower than that of p-cymene (39.75%) and thymoquinone (25.04%). In the agar dilution test, leaf and inflorescence EOs inhibited the growth of the six tested microorganisms at the concentration range of 0.156–0.625 µL/mL. Leaves collected at the vegetative phase of plant growth were characterised by the highest content of rosmarinic acid and didymin. Inflorescences harvested during the flowering of plants were rich in linarin. Flavonoid content was highly correlated with antioxidant activity of extracts. Due to these properties, *M. fistulosa* extracts and essential oils could be used in the food industry as natural preservatives or antioxidants, thereby contributing to the development of safer and more sustainable food products.

**Supplementary Information:**

The online version contains supplementary material available at 10.1007/s11130-024-01238-y.

## Introduction

*Monarda fistulosa* L. is one of several dozen species of *Monarda* L. naturally occurring in North America [1, 2]. Due to the high decorative and aromatic values, many varieties of this species have been cultivated also in Europe [3]. *M. fistulosa* was introduced to Europe in the seventeenth century as the first species from *Monarda* genus [4]. *M. fistulosa* is an erect, perennial, herbaceous plant with numerous shoots, reaching a height of 125 cm. Grey-green, lanceolate to ovate or deltoid-ovate leaves are decussately arranged on quadrangular stems. Branched inflorescence shoots have 5 to 9 inflorescences located individually on the tops of the main stem and axillary branches. Numerous bilabiate flowers are arranged in clusters above leafy bracts. The corolla is usually lilac in colour. However, across various cultivars, there are plants with a corolla colour from purple to white [1]. The common name of the species, wild bergamot, comes from the lemony, pungent scent resembling the scent of *Citrus bergamia* Risso & Poit [4].

*M. fistulosa* herb was used by Native American tribes to treat colds, catarrh, bronchial ailments, and to relieve headache and abdominal pain. The decoction of leaves has also been applied against acne. Due to its taste, it was used to flavour dishes and prepare drinks. The first European settlers used *M. fistulosa* to relieve nausea, vomiting, and as a carminative agent [1]. *M. fistulosa* herb contains a substantial amount of phenolic compounds and essential oil (EO), with the latter’s concentration reaching up to 4.83%. Components with well-documented antimicrobial potential (such as thymol and carvacrol) have been identified in the EO [5]. Significant quantity of thymoquinone, a compound with antioxidant, hepatoprotective, anti-inflammatory, and anti-cancer properties, has also been found [6]. It was the reason for initiating research on cultivation and post-harvest treatment of *M. fistulosa*, and isolation techniques allowing to increase the yield of this compound [7]. Among non-volatile compounds, phenolic acids (rosmarinic acid, caffeic acid, neochlorogenic acid, and chlorogenic acid) and flavonoids (luteolin, luteolin-7-O-glucoside, apigenin, apigenin-7-O-glucoside, acacetin-7-O-glucoside, isorhoifolin, linarin, didymin, and monardoside) have been identified in this plant [8, 9].

*M. fistulosa* can be consumed in several ways. Its leaves can be eaten raw or cooked, and its flowers can be used as an attractive edible garnish, while the entire plant above-ground part can serve as a potherb, a raw material for refreshing teas or a flavouring and health-promoting component of various dishes [9]. Raw material products from *Monarda*, e.g., *Monarda* leaves, are available on the market. However, they may originate from many species and cultivars from the chemically diverse *Monarda* genus. The introduction of quality standards for raw materials derived from this plant and used in the food industry can help increase customer confidence in the benefits offered by *M. fistulosa* and ensure repeatable and high quality of products.

Despite some existing literature on the composition and quantity of both EO and phenolics isolated from *M. fistulosa*, the variation in their accumulation across different plant parts at different phases of development remains unclear. The changes in the levels of the aforementioned groups of compounds may influence the biological activity of plant raw materials. Understanding the mechanisms that determine the content of the active components may help to obtain raw material of *M. fistulosa* of the desired quality in non-native climatic conditions.

In this context, the present study has two objectives. First, to assess the impact of the plant developmental phase on the quantity and quality of EOs and phenolics in the above-ground organs of *M. fistulosa* plants. Second, to determine the antimicrobial properties of the EOs and the antioxidant activity of extracts derived from these organs.

## Materials and methods

This section is presented in Supplementary Material (SM).

## Results and Discussion

### Content and Composition of Essential Oil

The quantity of essential oil (EO) present in the leaves and inflorescences varied depending on the phase of plant development (Table S1 in SM). In the leaves collected at the vegetative phase (phase 1) and at full flowering (phase 2) the content of EO was higher than at the fruit setting phase (phase 3): 4.03, 4.08, and 3.08%, respectively. A decline in the concentration of EO was also observed in the inflorescences: from 3.94% at the phase 2 to 1.06% at the phase 3. Malankina et al. [10] also noted a reduction in the content of EO in the above-ground parts of *M. fistulosa* plants after mass flowering: from 1.77% to less than two-thirds of that content at the end of flowering. The variation in the content of EO at different phases of phenology can be explained by the changing photosynthetic activity of plants, pressure of biotic and abiotic stress factors, changing density of secretory structures (with higher density of glandular trichomes usually correlated with higher essential oil content), as well as changes in the expression of genes and enzymes taking part in the secondary metabolism of plants [11–13].

Within the present study, a total of 55 volatile compounds were detected and identified, representing 98.00-99.87% of the composition of leaf and inflorescence EOs (Table S1 in SM). The impact of the plant phenological phase on the proportion of 16 compounds within the leaf EO and 27 compounds within the inflorescence EO was observed. In most of the analysed EOs, the main group of components were oxygenated monoterpenes, the content of which ranged from 45.41 to 69.69%. The EO extracted from inflorescences harvested at the phase 3 was distinguished by an elevated concentration of the monoterpene hydrocarbons (47.65%), corresponding to the high p-cymene content. The predominant constituents detected in the EO derived from the leaves were carvacrol (37.56%), p-cymene (17.71%), thymol (12.1%), and carvacrol methyl ether (5.13%). The main components of inflorescence EO were p-cymene (28.19%), carvacrol (27.89%), and thymoquinone (14.91%).

At the phase 2, the presence of geraniol in the EO of leaves (14.6%) and inflorescences (9.25%) was also observed. According to Mazza and Marshall’s [14] findings, the EO of certain *M. fistulosa* cultivars may contain over 90% of this compound. At the phase 3, the geraniol content and its derivatives in leaf and inflorescence EOs was negligible. This may suggest its further conversion to iridoid monoterpenoids, which was described as one of the metabolic pathways of geraniol synthesis [15]. However, the differences in the geraniol content depending on plant phenological phase were insignificant because of the large chemical variation among plants, which was reflected in high standard deviation values.

No significant effect of phenological phase on the carvacrol and thymol content in the leaf EO was observed either. The average thymol content was higher in the EO obtained from leaves (12.10%) than in the EO from inflorescences (2.47%). A significant influence of the phenological phase on the content of the main compounds in the EO obtained from inflorescences was observed. The content of p-cymene and thymoquinone increased significantly between the phases 2 and 3: from 16.63 to 39.75% and from 4.79 to 25.04%, respectively. In contrast, the carvacrol content dropped from 45.12 to 10.65%. Similar relationships have been found by Thompson et al. [16]. According to these authors, the contents of p-cymene, thymoquinone, and carvacrol in the EO from *M. fistulosa* senescent flowers were 37.3, 29.4, and 16.1%, respectively.

The thymoquinone biosynthesis pathway includes the prior conversion of p-cymene to thymol and/or carvacrol [17]. This would explain the reduction in carvacrol content, which was observed along with the increase in thymoquinone content. However, an increase in the content of p-cymene, a precursor of carvacrol and/or thymol, was also noted, which may suggest secondary formation of p-cymene that is not subject to further changes. A similar phenomenon was observed in other species of the *Lamiaceae* family [18]. The initial stages of *Thymus zygis* L. growth were distinguished by a significant biosynthesis of p-cymene, followed by a reduction in its content with a simultaneous increase in the content of thymol, and then an increase in the content of p-cymene [19]. Due to the diversity of inflorescence structures on which glandular trichomes occur, the biosynthesis pathway in these parts may be more complex than in leaves [20].

### Essential Oil Antimicrobial Activity

The EOs obtained from *M. fistulosa* plants harvested at the phase 2 had antimicrobial properties against four strains of foodborne pathogenic bacteria: *Escherichia coli*, *Campylobacter jejuni* subsp. *jejuni*, *Staphylococcus aureus*, *Listeria monocytogenes*, yeast *Candida albicans*, and mould *Aspergillus fumigatus* (Table S1 in SM). Leaf and inflorescence EOs inhibited the growth of all tested microorganisms at the concentration of 0.625 µL/mL. The most resistant strain, the growth of which was not inhibited by lower EO concentrations, was the Gram-negative bacterial strain of *C. jejuni*. In most of the tested strains, inhibition of microbial growth was observed at the EO concentration of 0.313 µL/mL. The EO from the inflorescences showed even higher antimicrobial activity, inhibiting the growth of *S. aureus* and *A. fumigatus* at the concentration of 0.156 µL/mL. This could be related to the slightly higher share of p-cymene and carvacrol in the EO, the compounds with well-documented antimicrobial activity [21, 22]. Thymoquinone content was also slightly higher in the inflorescence EO, but its relative percentage was much lower than that of carvacrol or p-cymene. Thymol does not appear to be responsible for the difference in antimicrobial activity of leaf and inflorescence EOs, either. Its content in the EOs subjected to the test was similar. In spite of slight differences in the composition of leaf and inflorescence EOs, they did not differ in the activity against four of the six microorganisms used in the study.

In the broth microdilution assay carried out by Ghabraie et al. [23], the minimum concentration of EO from *M. fistulosa* inflorescences inhibiting the growth of foodborne bacteria strains (*S. aureus*, *L. monocytogenes*, and *E. coli*) was in the range of 1.250–3.125 µL/mL. However, the plants used in the study differed in chemical type from those we examined, because of the high percentage of geraniol in EO (91.71%). In other studies, the activity of EO with a high thymol content was even lower, reaching a MIC value of 10 µL/mL [4]. Unlike the authors mentioned above, Inouye et al. [24] reported higher activity of *M. fistulosa* EO against the *C. albicans* strain. Comparing the results of antimicrobial activity tests is difficult due to the different research conditions and the chemical variability among the tested isolates obtained from genetically diverse plants [25]. Further studies employing multiple methods, including inhibition zone analysis, could provide a more comprehensive assessment of the potential applications of *M. fistulosa* EO as antimicrobial agents.

Standardisation of the raw material and selection of cultivated forms of *M. fistulosa* plants for industrial purposes may facilitate the identification of non-genetic factors influencing the quality and activity of the final product. This would increase the chances of introducing *M. fistulosa* raw materials to the market and using it on a larger scale, e.g. in the food industry to protect food products and their consumers from foodborne pathogens.

### Phenolic Compounds and Antioxidant Activity

Significant differences were observed in the total phenolic, total flavonoid content and antioxidant activity depending on the raw material (Table S1 in SM). Leaves were characterised by a higher content of total phenolics and total flavonoids than stems and inflorescences. The average contents of total phenolics in leaves, stems, and inflorescences were 3653.58, 2387.08 g, and 2028.23 mg GAE/100 g, respectively. The average total flavonoid contents in leaves, stems, and inflorescences were 8261.71 mg RUE/100 g, 4925.75 mg RUE/100 g, and 3906.98 mg RUE/100 g, respectively. Despite the fact that flavonoids belong to polyphenols, the results concerning total phenolics and total flavonoids cannot be compared with each other, mainly due to the different reagents used in these analyses.

The influence of the phenological phase on the total flavonoid content and antioxidant activity of different parts of the plant was observed. Total flavonoid content in the leaves collected at the phase 1 and at the phase 2 was similar, reaching 8860.81 and 8699.77 mg RUE/100 g, respectively. A reduction in the flavonoid content to 7224.55 mg RUE/100 g was observed at the phase 3. A similar phenomenon was observed in the case of inflorescences: the content of flavonoids decreased from 6126.58 mg RUE/100 g at the phase 2 to 1687.39 mg RUE/100 g at the phase 3. The inverse relationship was observed in the stems, where the flavonoid content gradually increased to 5843.92 mg RUE/100 g at the phase 3. The gradual accumulation of flavonoids in the stems was observed in the studies on *Rosmarinus officinalis* L [26]. The content of flavonoids previously reported in *M. fistulosa* herb was 5960–7680 mg/100 g expressed as isorhoifolin equivalent [8].

Total flavonoid content was strongly correlated with antioxidant activity (r^2^ = 0.989 for FRAP assay; r^2^ = 0.991 for DPPH assay). Slightly lower correlation was observed between total phenolic content and antioxidant activity in FRAP (r^2^ = 0.925) and DPPH assay (r^2^ = 0.906), as shown in Table S1 in SM. High correlation between total phenolic content and the results of antioxidant capacity assays was also found in the studies on lemon balm (*Melissa officinalis* L.) [27]. These studies also showed that the harvest date significantly impacted the total phenolic content and antioxidant activity. According to the literature, the antioxidant properties of plant materials are mainly related to the presence of phenolic compounds, but individual compounds and groups of phenolics show different strength of antioxidant activity, which is related to their structural features, including the number and position of hydroxyl groups and other substituents, and the presence of conjugated double bonds [28]. Among the phenolic compounds identified in the investigated *M. fistulosa* organs, the strongest correlation with antioxidant activity was found for luteolin-7-O-glucoside (r^2^ = 0.841 for FRAP assay; r^2^ = 0.838 for DPPH assay) and rosmarinic acid (r^2^ = 0.792 for FRAP assay; r^2^ = 0.801 for DPPH assay) (Table S4 in SM).

Rosmarinic acid was the dominant phenolic acid identified in *M. fisulosa* organs (Table S1 in SM). Its content in leaves ranged from 403.25 to 552.53 mg/100 g, and it was not significantly affected by the plant phenological phase. Rosmarinic acid content in inflorescences was lower and decreased during plant development. Conversely, in stems, the rosmarinic acid content increased and reached 327.3 mg/100 g at the phase 3. The differences in the accumulation of rosmarinic acid in various plant organs may be related to the differences in the expression of genes responsible for the production of enzymes involved in rosmarinic acid biosynthesis, different degradation rates, and transport of this compound [29]. A significant reduction in the content of most phenolic acids and flavonoids at the phase 3 in comparison to the phase 2 was observed in inflorescences. Linarin content was the highest in fully flowering inflorescences (677.34 mg/100 g) and it dropped drastically to 54.29 mg/100 g at the fruit setting phase. After the phase 2, a decrease in the content of other flavonoids (luteolin-7-O-glucoside, apigenin-7-O-glucoside, didymin, apigenin, narirutin, and prunin) in *M. fistulosa* inflorescences was also observed. In the leaves, the content of linarin and didymin was highest at the phase 1 (284.38 and 436.69 mg/100 g, respectively). In subsequent phenological phases, a decrease in the content of these compounds was observed. The content of apigenin and apigenin-7-O-glucoside in leaves slightly increased until the phase 2 and then decreased. The highest content of narirutin was observed in stems at the phase 3, reaching 214.8 mg/100 g. Stems were also the richest in prunin (420.45-532.66 mg/100 g).

The observed changes in the content of individual compounds in various organs depending on the phenological phase reflect the relationships described in the literature between various processes taking place in plants, such as biosynthesis, transport, gradual degradation of these compounds or their transformation into other metabolites during plant phenology [26]. A gradual decrease in the content of narirutin or rosmarinic acid in inflorescences, along with an increase in their concentration in stems, suggests the transport of these secondary metabolites. However, according to some authors, the transport of phenolic compounds via phloem is unlikely but possible in the case of small amounts and short distances for the purpose of fulfilling regulatory functions [30]. The diverse content of phenolic compounds, depending on the phenological phase and plant part, is related to the differences in organ-specific enzymatic activity and the expression of genes responsible for the synthesis of phenolic compounds [31]. Changes in their activity may be a response to biotic and abiotic stress factors [32].

The obtained results indicate that the content of phenolic compounds and the antioxidant potential were the highest in *M. fistulosa* leaves. Considering all aerial organs of this plant, the full flowering phase seems to be the most appropriate harvest term, as in the case of other plants from the *Lamiaceae* family [33, 34]. However, changes in the content of individual compounds in various parts of *M. fistulosa* plants depending on the phenological phase prompt further research on the mechanisms regulating the accumulation of secondary metabolites and create the possibility of adjusting the harvest date and method to the specific quality requirements. It is worth stressing that antioxidant activity of various plant organs and extracts prepared thereof may result not only from the presence of non-volatile phenolic compounds. Antioxidant properties have been reported for numerous EOs and their constituents, including those predominant in the studied *M. fistulosa* EOs: carvacrol, thymol, thymoquinone, and p-cymene [21, 35]. Further studies on antioxidant activity of essential oils obtained from this plant and the impact of their constituents on the antioxidant capacity of extracts would be recommended.

## Conclusion

The presented results indicate a high chemical variability of individual raw materials obtained from *M. fistulosa* depending on the phenological phase. The highest essential oil (EO) content in the above-ground part of the plant was observed at the full flowering phase. However, the EO obtained from the inflorescences collected during fruit setting contained significantly more thymoquinone. The antimicrobial activity exhibited by EOs from *M. fistulosa* leaves and inflorescences collected at the full flowering phase can be attributed to their high carvacrol content. Due to the demonstrated antimicrobial properties of these EOs against investigated foodborne pathogens, it seems justified to continue research on their possible use in the preservation of food products. The aerial parts of *M. fistulosa* were rich in rosmarinic acid, didymin, linarin, and prunin. Their content varied depending on the part of the plant and the phenological phase, influencing the antioxidant activity of the tested raw materials.

Leaves and inflorescences obtained from *M. fistulosa* plants can enrich the market of herbal raw materials for potential use in the food, pharmaceutical and cosmetic industries. Further investigation is needed, which could help standardise the raw material and define the conditions of its production and application.

## Electronic Supplementary Material

Below is the link to the electronic supplementary material.

Supplementary Material 1

## Data Availability

The datasets generated during and/or analysed during the current study are available from the corresponding author on reasonable request.
