# Supplementary material for: Influence of Plant Phenology on Chemical Composition of Monarda fistulosa L. Organs and their Bioactive Properties
Source: Plant Foods Hum Nutr. 2024 Sep 26;79(4):920–5. doi: 10.1007/s11130-024-01238-y (PMC11573850; doi:10.1007/s11130-024-01238-y)
Supplement: Supplementary file 1 — Supplementary Material 1 [file 11130_2024_1238_MOESM1_ESM.docx]

**SUPPLEMENTARY MATERIAL**

**Title:** Influence of Plant Phenology on Chemical Composition of *Monarda fistulosa* L. Organs and their Bioactive Properties

**Journal name:** Plant Foods for Human Nutrition

**Author Names:** Łukasz Gontar^*1^, Anna Geszprych^1^, Monika Sitarek-Andrzejczyk^2^, Ewa Osińska^1^

^1^Department of Vegetable and Medicinal Plants, Institute of Horticulture Sciences, Warsaw University of Life Sciences, 159 Nowoursynowska Street, 02-776, Warsaw, Poland

^2^Research and Innovation Centre Pro-Akademia, 9/11 Innowacyjna Street, 95-050, Konstantynów Łódzki, Poland

* Corresponding author: Łukasz Gontar. E-mail: gontar.lukasz@gmail.com

## Materials and Methods

### Plant Material

*M. fistulosa* seeds were purchased from Everwilde Farms (USA). Field trials were conducted between 2015 and 2017 on alluvial soil at the Experimental Field of the Department of Vegetable and Medicinal Plants, Warsaw University of Life Sciences (52°09'37.9"N 21°06'03.7"E). Preparation of the field, seedlings and post-harvest treatment of the collected raw materials were described previously [1]. Plants were harvested in the first week of July 2016 and 2017, at the phase of vegetative growth, before the emergence of flower buds (phase 1), in the first week of August 2016 and 2017, during flowering (phase 2), and in the second week of September 2016 and 2017, during fruit setting (phase 3) as shown in Fig. S1. The presented results are the averages of the two consecutive years.


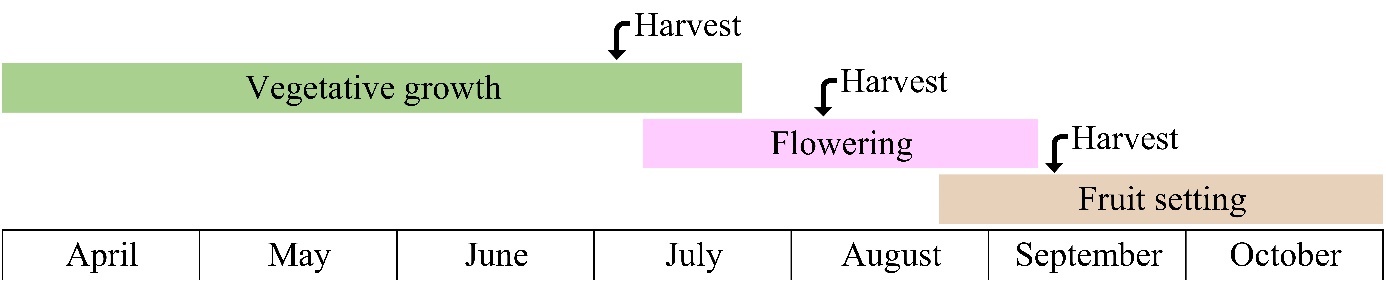


**Fig. S1** *Monarda fistulosa* phenology timeline with harvest periods in the second year of plant growth (2016 and 2017)

### Essential Oil Isolation

Essential oils (EOs) were obtained from *M. fistulosa* leaves and inflorescences using the hydrodistillation technique described in the Polish Pharmacopoeia [2] with modifications as presented previously [1]. Three biological replicates of each plant material were applied. The results were reported as mL/100 g of dry raw material. As the stems contained a low quantity of EO (less than 0.05 mL/100 g), they were excluded from subsequent analyses.

### Gas Chromatography-Mass Spectrometry (GC-MS)

The composition of EOs from *M. fistulosa* inflorescences and leaves collected at different phases of plant development were analysed using a gas chromatography-mass spectrometry (GC-MS) system. The conditions for separation and the method for identifying the separated compounds were previously described [1]. The system was equipped with a triple-axis detector (Agilent 7890A/5975C MSD, Agilent Technologies, Santa Clara, CA, USA) and a non-polar capillary column (HP-5MS 5% phenylmethylsiloxane; 30.00 m × 0.25 mm, 0.25 μm film thickness, Agilent Technologies, Santa Clara, CA, USA). The oven temperature was initially set at 50 °C for 5 minutes, followed by an increase of 3 °C per minute until reaching 240 °C, and then maintained for an additional minute. The injector temperature was maintained at 290 °C. Helium was used as the carrier gas at a flow rate of 0.8 mL per minute. One μL aliquots of the sample (comprising 5% essential oil in hexane) were introduced using a 7683B Injector in split mode (1:20). Electron ionisation was used for mass spectrometric detection, with ionisation energy set at 70 eV and a scanning range of 35 to 350 m/z. Data acquisition and analysis were conducted using the integrated data management software provided by the GC/MS manufacturer (Agilent ChemStation).

EO constituents were identified through comparative analysis of their mass spectra with reference spectra in the NIST 08 and Wiley 8th edition mass spectral libraries. Further confirmation of the mass spectra was achieved by comparing the constituents' calculated retention indices (RI), determined relative to a series of n-alkanes (C8–C20), with the RI values available in the NIST 08 mass spectral library.

### Testing of Antimicrobial Activity of Essential Oils

The EOs obtained from leaves and inflorescences of *M. fistulosa* collected at the phase 2 were subjected to antimicrobial activity assay (agar dilution method). The experiment was carried out with six microbial strains: *Candida albicans* (ATCC 10231) yeast, *Aspergillus fumigatus* (ATCC 204305) mould, and four bacteria: *Listeria monocytogenes* (ATCC 19111), *Staphylococcus aureus* (ATCC 25923), *Campylobacter jejuni* subsp. *jejuni* (ATCC 33560), and *Escherichia coli* (ATCC 25922). Cultures growth conditions were previously described [1]. Eight EO dilutions, ranging from 0.039 to 5 µL/mL, were prepared in triplicate. The EO was dissolved in sterile 20% dimethyl sulfoxide (Supelco, Bellefonte, PA, USA) in water. Aliquots (100 µL) of each EO solution was combined with 9.9 mL of the respective medium, vortexed, promptly dispensed onto a Petri dish, and allowed to solidify. Then, 100 µL aliquots of inoculum were spread onto the agar surface and incubated under the same conditions as the growth cultures. As positive controls, various concentrations of antibiotics – amphotericin, chloramphenicol, erythromycin, gentamicin, nystatin, and penicillin (Merck, Darmstadt, Germany) – were used. For negative controls, two solutions were applied: (i) 100 µL of 20% dimethyl sulfoxide (Supelco, Bellefonte, PA, USA) in water (used to dissolve the EOs) and (ii) 100 µL of 50% ethanol (Supelco, Bellefonte, PA, USA) in water (used to dissolve the antibiotics). The minimum inhibitory concentration (MIC) was defined as the lowest EO concentration at which the microbial strains' growth was visibly inhibited. The results were expressed as µL of EO per mL of medium (µL/mL).

### Extraction of Phenolic Compounds

Extraction was carried out with an ASE 350 apparatus (Thermo Scientific, Waltham, MA, USA). Stainless steel extraction cells (10 mL) were filled with cellulose filters and 0.5 g samples of powdered leaves, inflorescences, and stems. A detailed extraction procedure was previously described [1]. The extraction cell was filled with a solvent for each extraction procedure, pressurised to 1500 psi, and heated to reach the desired temperature of 100°C. Subsequently, the extraction process was carried out in three static cycles, each lasting 5 minutes. Following these cycles, the cell was flushed with a fresh aliquot of the extraction solvent, equivalent to 100% of the cell's volume, and then purged with a stream of nitrogen for 60 seconds. The extract was then transferred to a volumetric flask and filled up to 50 mL with 75% methanol in water.

The prepared extracts were used to determine the individual phenolic compounds, total phenolics, total flavonoids, and antioxidant activity of the investigated plant materials. Separate extracts were prepared from each biological replicate of every plant material.

### Determination of Total Phenolic and Total Flavonoid Contents

Total phenolic and total flavonoid contents were determined spectrophotometrically using Folin-Ciocalteu and AlCl_3_ reagents, respectively. In both assays, five-fold diluted extracts were used. The analytical procedure was described by Gontar et al. [1]. The absorbance was measured at 760 nm for total phenolic content determination and 510 nm for total flavonoid content determination using a UV-VIS spectrophotometer (UV-1800 spectrophotometer, Shimadzu, Kyoto, Japan). The results were calculated based on the established calibration curves of gallic acid and rutin (Merck, Darmstadt, Germany). The total phenolic content and total flavonoid content were expressed as gallic acid equivalent (GAE) and rutin equivalent (RUE), respectively, in mg/100 g of dry raw material.

### Antioxidant Activity Evaluation

The antioxidant activity of *M. fistulosa* extracts was determined using DPPH radical scavenging method and ferric reducing antioxidant power (FRAP) test, as described by Gontar et al. [1]. The absorbance was measured using a UV-1800 spectrophotometer (Shimadzu, Kyoto, Japan) at 517 nm and 593 nm in DPPH and FRAP assay, respectively. The results were calculated based on the calibration curves of Trolox (Merck, Darmstadt, Germany) and expressed as Trolox equivalent of antioxidant capacity (TEAC) in mg/100 g of dry raw material.

### HPLC Analysis of Phenolic Compounds

The analysis of *M. fistulosa* extracts was conducted using a Shimadzu Prominence HPLC system equipped with a photodiode array detector SPD-M20A, an autosampler SIL-20AC HT, and the LC solution 1.21 SP1 chromatography software. Chromatographic separation conditions were described previously [1]. For the gradient analysis, deionised water acidified to pH 3 using phosphoric acid (Supelco, Bellefonte, PA, USA) was used as mobile phase A, and acetonitrile (Supelco, Bellefonte, PA, USA) was used as mobile phase B. The elution gradient was set as follows: starting with 12.5% B at 0.01 min, increasing to 23% B at 4.00 min, reaching 50% B at 7.00 min, and then returning to 12.5% B at 7.01 min. A 5 µL aliquot of the filtered extract was injected and passed through a C18 reversed-phase column (Kinetex TM 2.6 µm; 100 mm × 4.60 mm; Phenomenex, Torrance, CA, USA), which was maintained at 40°C, with a flow rate set at 1.7 mL per minute. Phenolic compounds were identified by comparing their retention times and UV-VIS spectra with standards. The reference standards were purchased from Extrasynthese, Genay, France and Chromadex, Los Angeles, CA, USA. Data were collected at the following wavelengths: 255 nm (protocatechuic acid), 284 nm (narirutin, prunin, didymin), 309 nm (p-coumaric acid), 325 nm (chlorogenic acid, neochlorogenic acid, caffeic acid), 330 nm (rosmarinic acid), 335 nm (linarin, apigenin, apigenin-7-O-glucoside), 345 nm (luteolin-7-O-glucoside). The content of the determined compounds was calculated based on the calibration curves of the standards and the results were expressed in mg/100 g of dry raw material. Exemplary chromatograms of extracts obtained from leaves, stems, and inflorescences collected at the phase 2 are shown in Figures S2-S4.

**
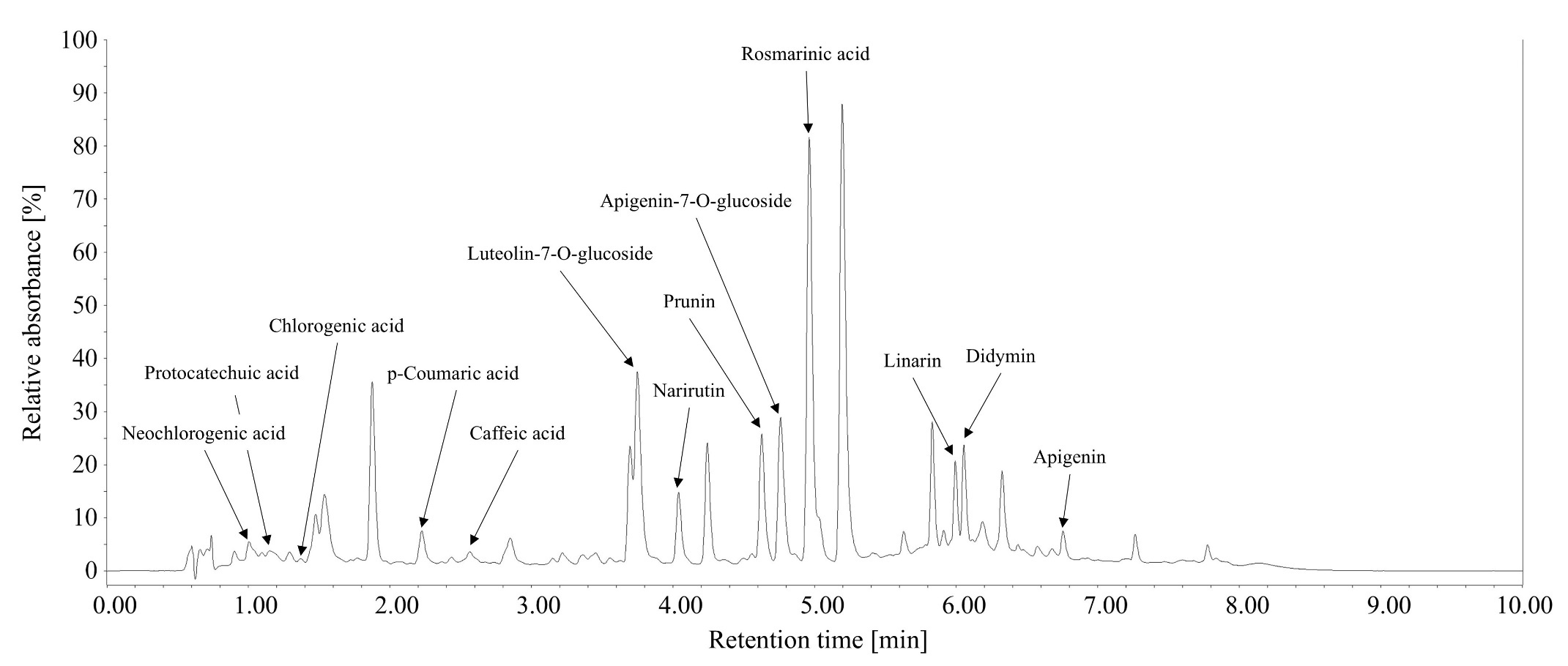
**

**Fig. S2** HPLC chromatogram of extract from *M. fisulosa* leaves collected at the full flowering phase (relative absorbance at 284 nm)


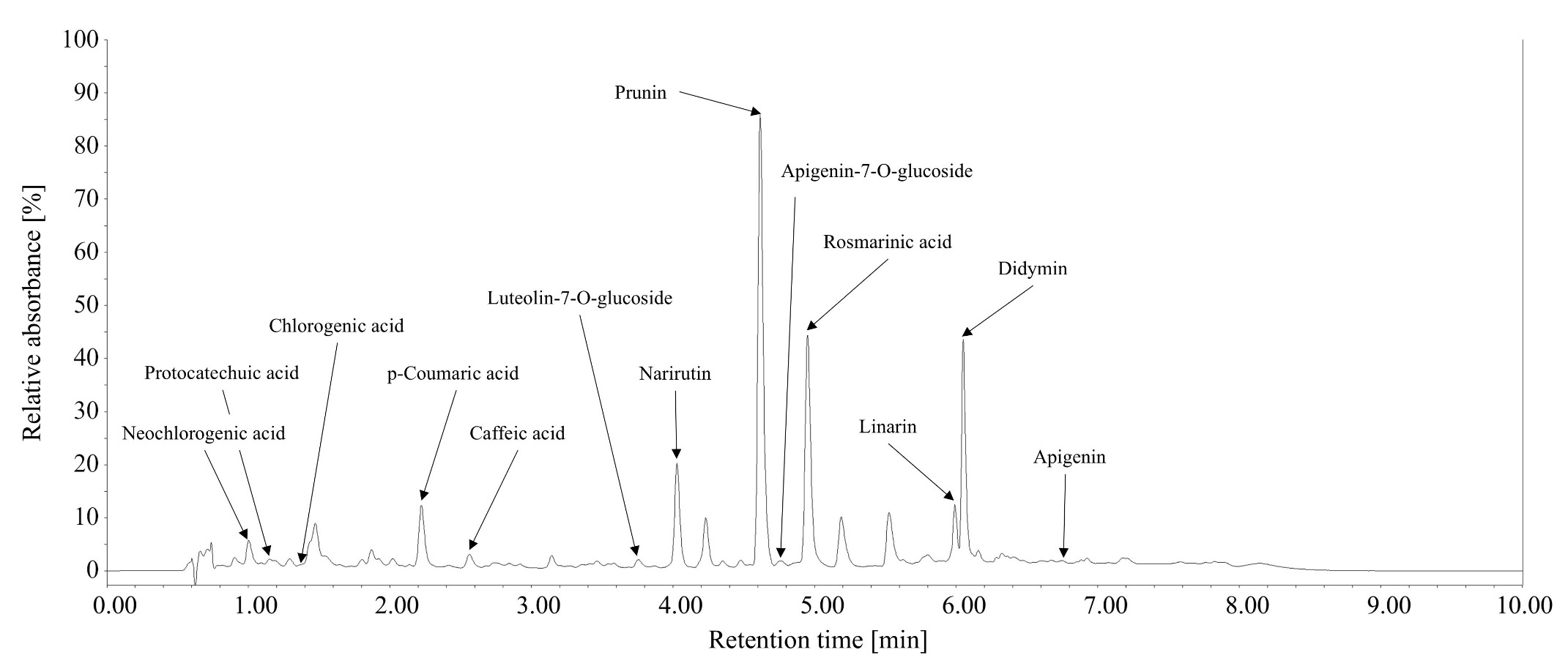


**Fig. S3** HPLC chromatogram of extract from *M. fistulosa* stems collected at the full flowering phase (relative absorbance at 284 nm)


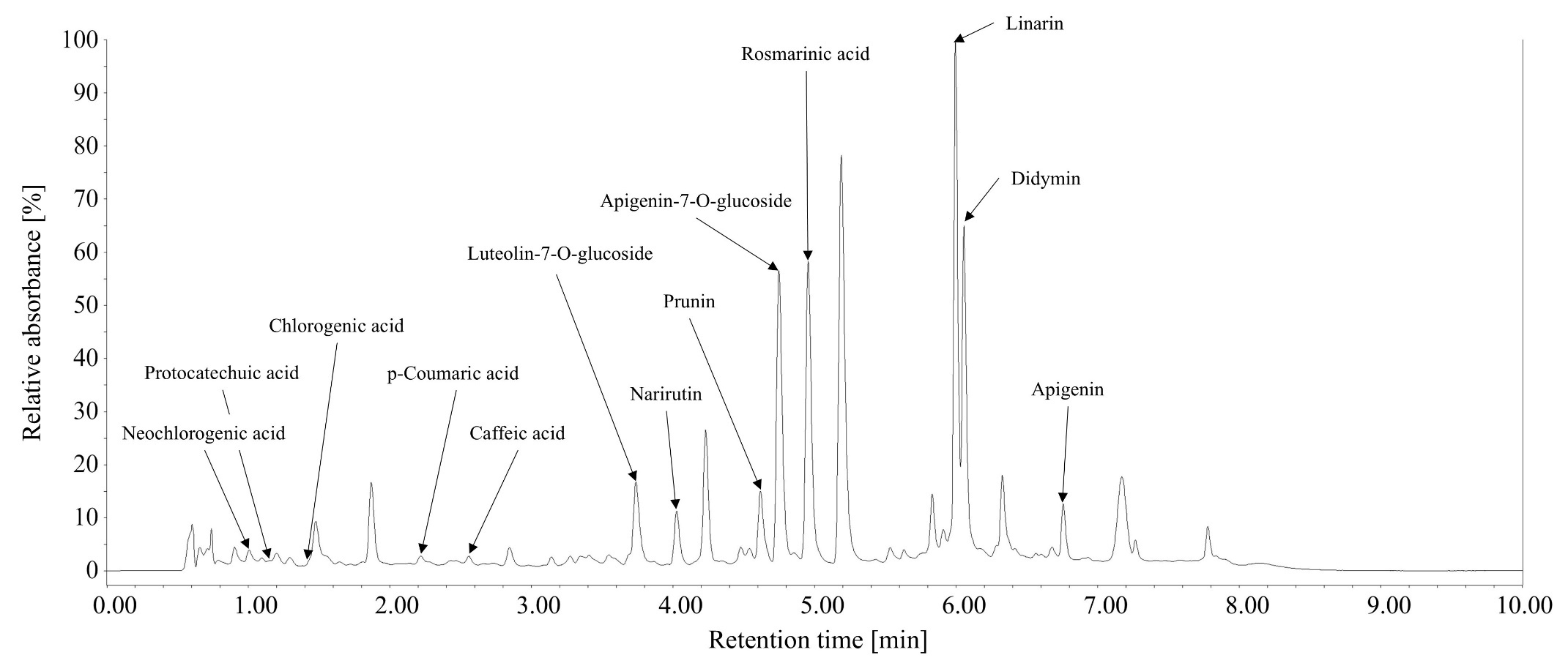


**Fig. S4** HPLC chromatogram of extract from *M. fistulosa* inflorescences collected at the full flowering phase (relative absorbance at 284 nm)

### Statistical Analysis

The data on the content of EO and its constituents, as well as the total and individual phenolics, and antioxidant activity, were analysed using one-way analysis of variance (ANOVA) with Statistica 14 software (TIBCO Software, Palo Alto, CA, USA). This was followed by the Tukey HSD test, with the significance level set at α=0.05. The way of comparing the results is explained under the Tables S1 and S3. Correlation analysis between the results regarding the content of phenolic compounds and antioxidant activity was performed using the same software.

**Table S1**

Essential oil composition (% area) and content (mL/100 g of dry material) in leaves and inflorescences of M. fistulosa collected at different phenological phases

| Compound | RI | RI_lit_ | Leaves | | | | Inflorescences | | |
| --- | --- | --- | --- | --- | --- | --- | --- | --- | --- |
|  |  |  | Vegetative growth (phase 1) | Full flowering  (phase 2) | Fruit setting  (phase 3) | Mean | Full flowering  (phase 2) | Fruit setting  (phase 3) | Mean |
| α-Thujene | 925 | 924 | 1.86 ± 0.41 ^a^ | 1.23 ± 0.34 ^b^ | 1.44 ± 0.32 ^ab^ | 1.51 ± 0.45 | 1.65 ± 0.64 | 1.35 ± 0.23 | 1.50 ± 0.50 |
| α-Pinene | 930 | 932 | 0.67 ± 0.11 ^a^ | 0.45 ± 0.14 ^b^ | 0.46 ± 0.10 ^b^ | 0.53 ± 0.16 | 0.54 ± 0.22 | 0.64 ± 0.16 | 0.59 ± 0.20 |
| 2,4-Thujadiene | 942 | 953 | 0.01 ± 0.01 ^b^ | 0.02 ± 0.01 ^b^ | 0.04 ± 0.01 ^a^ | 0.02 ± 0.01 ^B^ | 0.00 ± 0.00 ^b^ | 0.12 ± 0.03 ^a^ | 0.06 ± 0.06 ^A^ |
| Camphene | 944 | 946 | 0.14 ± 0.04 | 0.10 ± 0.04 | 0.08 ± 0.01 | 0.11 ± 0.04 | 0.10 ± 0.04 | 0.16 ± 0.06 | 0.13 ± 0.06 |
| Sabinene | 970 | 969 | 0.07 ± 0.06 | 0.06 ± 0.02 | 0.05 ± 0.02 | 0.06 ± 0.04 ^B^ | 0.07 ± 0.02 ^b^ | 0.12 ± 0.02 ^a^ | 0.09 ± 0.03 ^A^ |
| β-Pinene | 972 | 974 | 0.16 ± 0.05 ^a^ | 0.10 ± 0.04 ^b^ | 0.10 ± 0.02 ^b^ | 0.12 ± 0.05 | 0.11 ± 0.05 | 0.12 ± 0.03 | 0.12 ± 0.04 |
| 1-Octen-3-ol | 980 | 974 | 3.33 ± 0.91 | 2.83 ± 0.70 | 3.28 ± 1.21 | 3.15 ± 0.99 ^A^ | 1.86 ± 0.35 | 2.52 ± 0.79 | 2.19 ± 0.70 ^B^ |
| 3-Octanone | 987 | 979 | 0.04 ± 0.01 ^b^ | 0.08 ± 0.01 ^b^ | 0.14 ± 0.06 ^a^ | 0.09 ± 0.06 | 0.02 ± 0.01 ^b^ | 0.11 ± 0.05 ^a^ | 0.07 ± 0.06 |
| β-Myrcene | 991 | 988 | 0.04 ± 0.05 | 0.41 ± 0.40 | 0.36 ± 0.18 | 0.27 ± 0.31 | 0.22 ± 0.21 | 0.08 ± 0.03 | 0.15 ± 0.16 |
| 3-Octanol | 997 | 988 | 0.10 ± 0.04 | 0.10 ± 0.01 | 0.11 ± 0.05 | 0.10 ± 0.04 | 0.05 ± 0.01 ^b^ | 0.11 ± 0.05 ^a^ | 0.08 ± 0.05 |
| α-Phellandrene | 1002 | 1002 | 0.09 ± 0.06 ^b^ | 0.13 ± 0.06 ^ab^ | 0.19 ± 0.02 ^a^ | 0.14 ± 0.07 | 0.12 ± 0.04 ^a^ | 0.07 ± 0.02 ^b^ | 0.09 ± 0.04 |
| δ-3-Carene | 1007 | 1008 | 0.21 ± 0.05 ^a^ | 0.14 ± 0.04 ^b^ | 0.16 ± 0.03 ^ab^ | 0.17 ± 0.05 | 0.17 ± 0.05 | 0.17 ± 0.02 | 0.17 ± 0.04 |
| α-Terpinene | 1015 | 1014 | 3.26 ± 1.03 ^a^ | 1.94 ± 0.49 ^b^ | 2.74 ± 0.34 ^ab^ | 2.64 ± 0.87 | 2.81 ± 0.53 ^a^ | 1.16 ± 0.41 ^b^ | 1.98 ± 0.95 |
| p-Cymene | 1025 | 1020 | 18.09 ± 3.05 | 14.91 ± 3.41 | 20.14 ± 4.79 | 17.71 ± 4.39 ^B^ | 16.63 ± 2.30 ^b^ | 39.75 ± 6.13 ^a^ | 28.19 ± 12.45 ^A^ |
| Limonene | 1027 | 1024 | 2.81 ± 1.74 | 2.04 ± 1.12 | 1.15 ± 0.40 | 2.00 ± 1.39 | 2.02 ± 1.08 | 0.91 ± 0.75 | 1.47 ± 1.09 |
| 1,8-Cineole | 1029 | 1026 | 0.01 ± 0.02 | 0.01 ± 0.01 | 0.01 ± 0.02 | 0.01 ± 0.02 | 0.01 ± 0.02 | 0.00 ± 0.00 | 0.01 ± 0.01 |
| γ-Terpinene | 1058 | 1054 | 0.05 ± 0.06 | 1.35 ± 1.67 | 1.10 ± 1.10 | 0.83 ± 1.28 | 0.28 ± 0.39 | 0.19 ± 0.06 | 0.24 ± 0.28 |
| *cis*-Sabinene hydrate | 1065 | 1065 | 0.78 ± 0.11 | 0.73 ± 0.18 | 0.91 ± 0.19 | 0.81 ± 0.18 ^B^ | 0.67 ± 0.15 ^b^ | 2.16 ± 0.24 ^a^ | 1.42 ± 0.77 ^A^ |
| 1-Nonen-3-ol | 1081 | 1081 | 0.07 ± 0.02 ^ab^ | 0.05 ± 0.01 ^b^ | 0.08 ± 0.01 ^a^ | 0.07 ± 0.02 | 0.04 ± 0.01 ^b^ | 0.10 ± 0.02 ^a^ | 0.07 ± 0.04 |
| p-Cymenene | 1087 | 1089 | 0.09 ± 0.03 ^b^ | 0.10 ± 0.03 ^b^ | 0.16 ± 0.05 ^a^ | 0.12 ± 0.05 | 0.08 ± 0.02 ^b^ | 0.28 ± 0.07 ^a^ | 0.18 ± 0.11 |
| *trans*-Sabinene hydrate | 1096 | 1098 | 0.12 ± 0.06 | 0.13 ± 0.02 | 0.12 ± 0.03 | 0.12 ± 0.04 ^B^ | 0.13 ± 0.03 ^b^ | 0.37 ± 0.02 ^a^ | 0.25 ± 0.13 ^A^ |
| Linalool | 1100 | 1095 | 0.13 ± 0.03 | 0.24 ± 0.22 | 0.05 ± 0.03 | 0.14 ± 0.15 | 0.24 ± 0.31 | 0.03 ± 0.01 | 0.13 ± 0.24 |

**Table S1** continued

| Compound | RI | RI_lit_ | Leaves | | | | Inflorescences | | |
| --- | --- | --- | --- | --- | --- | --- | --- | --- | --- |
|  |  |  | Vegetative growth (phase 1) | Full flowering  (phase 2) | Fruit setting  (phase 3) | Mean | Full flowering  (phase 2) | Fruit setting  (phase 3) | Mean |
| Nonanal | 1105 | 1100 | 0.02 ± 0.01 | 0.08 ± 0.05 | 0.20 ± 0.19 | 0.10 ± 0.14 | 0.01 ± 0.01 ^b^ | 0.47 ± 0.30 ^a^ | 0.24 ± 0.32 |
| Oct-1-en-3-yl acetate | 1115 | 1110 | 0.00 ± 0.00 | 0.00 ± 0.01 | 0.01 ± 0.01 | 0.00 ± 0.01 ^B^ | 0.00 ± 0.00 ^b^ | 0.05 ± 0.01 ^a^ | 0.02 ± 0.02 ^A^ |
| *cis*-Limonene oxide | 1132 | 1132 | 0.13 ± 0.05 ^a^ | 0.04 ± 0.03 ^b^ | 0.04 ± 0.01 ^b^ | 0.07 ± 0.06 | 0.09 ± 0.04 | 0.07 ± 0.03 | 0.08 ± 0.04 |
| *trans*-Verbenol | 1143 | 1140 | 0.00 ± 0.00 | 0.00 ± 0.01 | 0.00 ± 0.00 | 0.00 ± 0.01 ^B^ | 0.00 ± 0.00 ^b^ | 0.14 ± 0.02 ^a^ | 0.07 ± 0.07 ^A^ |
| Borneol | 1163 | 1165 | 0.22 ± 0.14 | 0.16 ± 0.14 | 0.11 ± 0.02 | 0.16 ± 0.13 | 0.16 ± 0.10 | 0.19 ± 0.03 | 0.18 ± 0.07 |
| Terpinene-4-ol | 1175 | 1174 | 0.67 ± 0.14 | 0.48 ± 0.12 | 0.65 ± 0.19 | 0.60 ± 0.17 | 0.64 ± 0.14 | 0.57 ± 0.14 | 0.60 ± 0.14 |
| p-Cymene-8-ol | 1184 | 1179 | 0.05 ± 0.02 | 0.13 ± 0.07 | 0.22 ± 0.28 | 0.13 ± 0.18 ^B^ | 0.03 ± 0.02 ^b^ | 0.65 ± 0.20 ^a^ | 0.34 ± 0.34 ^A^ |
| α-Terpineol | 1188 | 1186 | 0.02 ± 0.02 ^b^ | 0.04 ± 0.04 ^ab^ | 0.12 ± 0.08 ^a^ | 0.06 ± 0.07 | 0.09 ± 0.06 | 0.11 ± 0.02 | 0.10 ± 0.05 |
| Myrtenol | 1193 | 1194 | 0.09 ± 0.06 | 0.03 ± 0.04 | 0.04 ± 0.04 | 0.05 ± 0.05 | 0.01 ± 0.02 ^b^ | 0.07 ± 0.03 ^a^ | 0.04 ± 0.04 |
| Cumin aldehyde | 1238 | 1238 | 0.00 ± 0.00 | 0.14 ± 0.19 | 0.09 ± 0.08 | 0.08 ± 0.13 ^B^ | 0.08 ± 0.13 ^b^ | 0.60 ± 0.25 ^a^ | 0.34 ± 0.33 ^A^ |
| Carvacrol methyl ether | 1244 | 1241 | 3.67 ± 2.32 | 6.42 ± 5.39 | 5.31 ± 3.73 | 5.13 ± 4.17 | 3.34 ± 2.21 | 5.76 ± 4.60 | 4.55 ± 3.80 |
| Thymoquinone | 1249 | 1248 | 6.77 ± 5.49 | 2.05 ± 1.86 | 1.54 ± 0.86 | 3.45 ± 4.12 ^B^ | 4.79 ± 3.14 ^b^ | 25.04 ± 4.15 ^a^ | 14.91 ± 10.77 ^A^ |
| Geraniol | 1257 | 1249 | 0.06 ± 0.14 | 14.60 ± 16.39 | 0.07 ± 0.15 | 4.91 ± 11.68 | 9.25 ± 14.85 | 0.00 ± 0.00 | 4.63 ± 11.47 |
| Bornyl acetate | 1286 | 1287 | 0.00 ± 0.00 | 0.03 ± 0.04 | 0.00 ± 0.00 | 0.01 ± 0.03 ^B^ | 0.02 ± 0.03 ^b^ | 0.19 ± 0.13 ^a^ | 0.11 ± 0.13 ^A^ |
| Thymol | 1299 | 1289 | 14.09 ± 12.25 | 4.75 ± 6.92 | 17.45 ± 10.65 | 12.10 ± 11.52 ^A^ | 3.97 ± 7.17 | 0.97 ± 0.69 | 2.47 ± 5.31 ^B^ |
| Carvacrol | 1305 | 1298 | 36.64 ± 11.66 | 39.75 ± 13.00 | 36.28 ± 5.96 | 37.56 ± 10.77 | 45.12 ± 10.5 ^a^ | 10.65 ± 4.92 ^b^ | 27.89 ± 19.09 |
| α-Cubebene | 1349 | 1345 | 0.04 ± 0.02 | 0.04 ± 0.01 | 0.03 ± 0.01 | 0.04 ± 0.01 ^A^ | 0.03 ± 0.01 ^a^ | 0.01 ± 0.01 ^b^ | 0.02 ± 0.01 ^B^ |
| Eugenol | 1358 | 1356 | 0.07 ± 0.05 | 0.04 ± 0.04 | 0.01 ± 0.01 | 0.04 ± 0.05 | 0.03 ± 0.03 | 0.00 ± 0.00 | 0.02 ± 0.03 |
| α-Copaene | 1374 | 1374 | 0.14 ± 0.03 ^a^ | 0.11 ± 0.02 ^ab^ | 0.10 ± 0.01 ^b^ | 0.12 ± 0.03 | 0.10 ± 0.01 | 0.11 ± 0.03 | 0.10 ± 0.02 |
| β-Bourbonene | 1383 | 1387 | 0.14 ± 0.03 ^ab^ | 0.17 ± 0.04^a^ | 0.11 ± 0.02 ^b^ | 0.14 ± 0.04 ^A^ | 0.05 ± 0.01 | 0.05 ± 0.01 | 0.05 ± 0.01 ^B^ |
| Caryophyllene | 1417 | 1417 | 0.73 ± 0.29 | 0.57 ± 0.14 | 0.44 ± 0.12 | 0.58 ± 0.23 | 0.60 ± 0.15 | 0.39 ± 0.15 | 0.50 ± 0.18 |
| β-Copaene | 1426 | 1430 | 0.07 ± 0.02 | 0.07 ± 0.02 | 0.06 ± 0.01 | 0.07 ± 0.02 ^A^ | 0.05 ± 0.02 | 0.04 ± 0.01 | 0.04 ± 0.01 ^B^ |
| α-Humulene | 1450 | 1452 | 0.07 ± 0.03 | 0.05 ± 0.01 | 0.06 ± 0.01 | 0.06 ± 0.02 | 0.03 ± 0.01 ^b^ | 0.08 ± 0.03 ^a^ | 0.06 ± 0.03 |

**Table S1** continued

| Compound | RI | RI_lit_ | Leaves | | | | Inflorescences | | |
| --- | --- | --- | --- | --- | --- | --- | --- | --- | --- |
|  |  |  | Vegetative growth (phase 1) | Full flowering  (phase 2) | Fruit setting  (phase 3) | Mean | Full flowering  (phase 2) | Fruit setting  (phase 3) | Mean |
| γ-Muurolene | 1475 | 1478 | 0.17 ± 0.06 | 0.10 ± 0.04 | 0.11 ± 0.03 | 0.13 ± 0.05 ^A^ | 0.11 ± 0.04 ^a^ | 0.05 ± 0.02 ^b^ | 0.08 ± 0.04 ^B^ |
| Germacrene D | 1479 | 1484 | 1.11 ± 0.56 | 1.00 ± 0.37 | 0.70 ± 0.17 | 0.94 ± 0.43 | 1.01 ± 0.27 ^a^ | 0.50 ± 0.07 ^b^ | 0.76 ± 0.33 |
| β-Selinene | 1483 | 1489 | 0.07 ± 0.02 ^a^ | 0.03 ± 0.02 ^b^ | 0.04 ± 0.01 ^b^ | 0.05 ± 0.02 ^A^ | 0.03 ± 0.01 | 0.02 ± 0.02 | 0.03 ± 0.02 ^B^ |
| α-Selinene | 1492 | 1498 | 0.14 ± 0.03 ^a^ | 0.08 ± 0.04 ^b^ | 0.09 ± 0.03 ^ab^ | 0.10 ± 0.04 ^A^ | 0.10 ± 0.04 ^a^ | 0.04 ± 0.01 ^b^ | 0.07 ± 0.04 ^B^ |
| α-Muurolene | 1499 | 1500 | 0.06 ± 0.02 | 0.08 ± 0.09 | 0.07 ± 0.08 | 0.07 ± 0.07 | 0.04 ± 0.01 | 0.34 ± 0.32 | 0.19 ± 0.27 |
| γ-Cadinene | 1512 | 1513 | 0.14 ± 0.04 | 0.10 ± 0.04 | 0.10 ± 0.03 | 0.11 ± 0.04 | 0.10 ± 0.04 | 0.06 ± 0.03 | 0.08 ± 0.04 |
| δ-Cadinene | 1522 | 1522 | 0.32 ± 0.10 | 0.21 ± 0.07 | 0.21 ± 0.06 | 0.25 ± 0.10 ^A^ | 0.25 ± 0.08 ^a^ | 0.07 ± 0.03 ^b^ | 0.16 ± 0.11 ^B^ |
| Thymohydroquinone | 1556 | 1553 | 1.12 ± 0.61 | 0.78 ± 0.51 | 2.05 ± 2.21 | 1.32 ± 1.46 | 1.07 ± 0.48 ^a^ | 0.37 ± 0.21 ^b^ | 0.72 ± 0.51 |
| Caryophyllene oxide | 1580 | 1582 | 0.05 ± 0.03 | 0.05 ± 0.03 | 0.06 ± 0.02 | 0.05 ± 0.03 | 0.02 ± 0.01 | 0.31 ± 0.30 | 0.17 ± 0.26 |
| α-Cadinol | 1652 | 1652 | 0.05 ± 0.03 | 0.03 ± 0.02 | 0.06 ± 0.05 | 0.04 ± 0.04 | 0.03 ± 0.02 | 0.09 ± 0.07 | 0.06 ± 0.06 |
| Monoterpene hydrocarbons | | | 28.47 ± 3.86 | 23.83 ± 5.87 | 29.20 ± 5.10 | 27.17 ± 5.55 ^B^ | 25.61 ± 3.94 ^b^ | 47.65 ± 6.60 ^a^ | 36.63 ± 12.29 ^A^ |
| Oxygenated monoterpenes | | | 63.74 ± 4.97 | 69.69 ± 5.78 | 64.06 ± 5.83 | 65.83 ± 6.18 ^A^ | 68.95 ± 4.20 ^a^ | 45.41 ± 6.61 ^b^ | 57.18 ± 13.01 ^B^ |
| Sesquiterpene hydrocarbons | | | 3.21 ± 0.71 ^a^ | 2.60 ± 0.47 ^ab^ | 2.13 ± 0.29 ^b^ | 2.65 ± 0.68 | 2.52 ± 0.35 ^a^ | 1.77 ± 0.57 ^b^ | 2.15 ± 0.60 |
| Oxygenated sesquiterpenes | | | 0.09 ± 0.04 | 0.08 ± 0.04 | 0.12 ± 0.07 | 0.10 ± 0.06 | 0.05 ± 0.03 ^b^ | 0.41 ± 0.30 ^a^ | 0.23 ± 0.28 |
| Others | | | 3.56 ± 0.91 | 3.14 ± 0.70 | 3.83 ± 1.27 | 3.51 ± 1.03 ^A^ | 1.97 ± 0.36 ^b^ | 3.37 ± 0.89 ^a^ | 2.67 ± 0.97 ^B^ |
| Sum of identified compounds | | | 99.08 ± 0.48 | 99.34 ± 0.40 | 99.33 ± 0.41 | 99.25 ± 0.45 | 99.10 ± 0.22 | 98.62 ± 0.46 | 98.86 ± 0.43 |
| Essential oil content | | | 4.03 ± 0.43 ^a^ | 4.08 ± 0.30 ^a^ | 3.08 ± 0.45 ^b^ | 3.71 ± 0.60 ^A^ | 3.94 ± 0.76 ^a^ | 1.06 ± 0.16 ^b^ | 2.50 ± 1.55 ^B^ |
| The reported values represent the means of three independent replicates in each experimental year (2016, 2017), with standard deviations (± SD) provided. However, the values presented in the last column for each raw material (leaves and inflorescences) represent the means derived from all replicates across all phenological phases and experimental years for that specific raw material, also accompanied by standard deviations (± SD). The following pairwise comparisons were performed within each row:   1. For a single raw material (leaves or inflorescences), means from different plant growth phases marked with different lowercase letters (e.g. a, b) differ significantly according to the Tukey test at the significance level α=0.05. 2. For different raw materials (leaves and inflorescences), means marked with different uppercase letters (e.g. A, B) differ significantly according to the Tukey test at the significance level α=0.05.   RI_calc._ – Retention indices determined on the HP-5MS column.  RI_lit._ – Retention indices according to literature [3]. | | | | | | | | | |

**Table S2**

Minimum inhibitory concentration (MIC) of essential oils obtained from leaves and inflorescences of M. fistulosa collected at the full flowering phase (µL/mL) and MIC of positive controls (antibiotics) (µg/mL)

| Microorganism | Essential oil (µL/mL) | | Antibiotics (µg/mL) | | | | | |
| --- | --- | --- | --- | --- | --- | --- | --- | --- |
|  | Leaves | Inflorescences | Amphotericin | Chloramphenicol | Erythromycin | Gentamicin | Nystatin | Penicillin |
| *Staphylococcus aureus* ATCC 25923 | 0.313 | 0.156 | - | 4 | 2 | 2 | - | 0.5 |
| *Escherichia coli* ATCC 25922 | 0.313 | 0.313 | - | 8 | 128 | 16 | - | 128 |
| *Listeria monocytogenes* ATCC 19111 | 0.313 | 0.313 | - | 8 | 0.5 | 0.5 | - | 0.5 |
| *Campylobacter jejuni* subsp. *jejuni* ATCC 33560 | 0.625 | 0.625 | - | 4 | 16 | 1 | - | 8 |
| *Aspergillus fumigatus* ATCC 204305 | 0.313 | 0.156 | 4 | - | - | - | 4 | - |
| *Candida albicans* ATCC 10231 | 0.313 | 0.313 | 0.5 | - | - | - | 2 | - |
| (-) antimicrobial activity was not observed at the tested concentrations of antibiotics ranging from 0.5 to 128 µg/mL | | | | | | | | |

**Table S3**

Total phenolic and total flavonoid contents, antioxidant capacity, and individual phenolic contents (mg/100 g of dry raw material) of various organs of M. fistulosa at different phenological phases

| Assay | Leaves | | | | Stems | | | | Inflorescences | | |
| --- | --- | --- | --- | --- | --- | --- | --- | --- | --- | --- | --- |
|  | Vegetative growth (phase 1) | Full flowering (phase 2) | Fruit setting (phase 3) | Mean | Vegetative growth (phase 1) | Full flowering (phase 2) | Fruit setting (phase 3) | Mean | Full flowering (phase 2) | Fruit setting (phase 3) | Mean |
| Total phenolic content  (gallic acid equivalent) | 3908.96  ± 491.08 | 3792.29  ± 722.62 | 3259.48  ± 458.06 | 3653.58  ± 635.86 ^A^ | 2118.33  ± 576.19 | 2326.67  ± 427.10 | 2716.25  ± 478.54 | 2387.08  ± 556.07 ^B^ | 3015.21  ± 1069.92 ^a^ | 1041.25  ± 519.64 ^b^ | 2028.23  ± 1296.73 ^B^ |
| Total flavonoid content  (rutin equivalent) | 8860.81  ± 695.14 ^a^ | 8699.77  ± 692.66 ^a^ | 7224.55  ± 704.77 ^b^ | 8261.71  ± 1014.27 ^A^ | 4087.16  ± 1452.79 ^b^ | 4846.17  ± 561.06 ^ab^ | 5843.92  ± 831.74 ^a^ | 4925.75  ± 1247.63 ^B^ | 6126.58  ± 1858.48 ^a^ | 1687.39  ± 427.10 ^b^ | 3906.98  ± 2597.07 ^B^ |
| FRAP  (Trolox equivalent) | 5393.28  ± 315.46 ^a^ | 5476.61  ± 695.14 ^a^ | 4307.26  ± 462.28 ^b^ | 5059.05  ± 741.10 ^A^ | 2523.66  ± 821.22 ^b^ | 2937.63  ± 339.91 ^ab^ | 3530.38  ± 478.09 ^a^ | 2997.22  ± 714.28 ^B^ | 3891.94  ± 1350.78 ^a^ | 967.20  ± 309.85 ^b^ | 2429.57  ± 1760.35 ^B^ |
| DPPH  (Trolox equivalent) | 3595.27  ± 285.07 ^a^ | 3569.14  ± 375.48 ^a^ | 2919.03  ± 322.34 ^b^ | 3361.14  ± 454.49 ^A^ | 1823.74  ± 561.56 ^b^ | 2087.73  ± 193.02 ^ab^ | 2497.70  ± 341.64 ^a^ | 2136.39  ± 483.04 ^B^ | 2625.30  ± 810.15 ^a^ | 1023.53  ± 208.60 ^b^ | 1824.41  ± 995.67 ^B^ |
| Neochlorogenic acid | 23.49  ± 10.88 | 28.38  ± 11.65 | 33.78  ± 5.15 | 28.55  ± 10.54 ^A^ | 29.26  ± 12.75 ^ab^ | 20.67  ± 7.40 ^b^ | 49.01  ± 19.74 ^a^ | 32.98  ± 18.52 ^A^ | 14.53  ± 4.33 ^a^ | 2.66  ± 0.49 ^b^ | 8.60  ± 6.69 ^B^ |
| Protocatechuic acid | 13.97  ± 1.34 | 14.31  ± 1.80 | 13.19  ± 1.24 | 13.82  ± 1.55 | 12.84  ± 0.21 | 12.62  ± 0.11 | 13.76  ± 1.57 | 13.07  ± 1.04 | 14.56  ± 1.83 | 12.77  ± 0.27 | 13.67  ± 1.58 |
| Chlorogenic acid | 10.45  ± 3.17 ^b^ | 7.86  ± 3.00 ^b^ | 20.27  ± 8.74 ^a^ | 12.86  ± 7.77 ^AB^ | 11.41  ± 4.39 ^b^ | 7.96  ± 5.34 ^b^ | 26.39  ± 10.68 ^a^ | 15.25  ± 10.86 ^A^ | 15.03  ± 4.15 ^a^ | 0.00  ± 0.00 ^b^ | 6.22  ± 9.29 ^B^ |
| p-Coumaric acid | 17.78  ± 4.36 | 10.87  ± 3.83 | 13.66  ± 4.56 | 14.11  ± 5.12 ^A^ | 14.24  ± 7.33 | 10.83  ± 6.64 | 15.04  ± 11.33 | 13.37  ± 8.87 ^A^ | 3.75  ± 1.30 ^a^ | 0.22  ± 0.23 ^b^ | 1.98  ± 2.00 ^B^ |
| Caffeic acid | 3.19  ± 1.18 | 3.38  ± 1.39 | 4.42  ± 3.90 | 3.66  ± 2.54 | 1.33  ± 0.63 | 1.51  ± 0.64 | 3.12  ± 2.01 | 1.99  ± 1.50 | 1.66  ± 2.12 | 1.85  ± 2.94 | 1.75  ± 2.57 |
| Luteolin-7-O-glucoside | 222.11  ± 45.45 | 216.13  ± 69.00 | 138.11  ± 39.03 | 192.12  ± 65.17 ^A^ | 11.02  ± 3.29 | 10.62  ± 5.66 | 32.73  ± 27.58 | 18.13  ± 19.35 ^C^ | 133.16  ± 39.49 ^a^ | 9.21  ± 4.71 ^b^ | 71.18  ± 68.05 ^B^ |
| Narirutin | 92.27  ± 8.91 | 92.74  ± 17.68 | 77.49  ± 13.76 | 87.50  ± 15.61 ^B^ | 98.96  ± 57.59 ^b^ | 99.71  ± 25.12 ^b^ | 214.80  ± 57.31 ^a^ | 137.82  ± 73.30 ^A^ | 76.14  ± 29.23 ^a^ | 22.80  ± 10.25^b^ | 49.47  ± 34.51 ^B^ |

**Table S3** continued

| Assay | Leaves | | | | Stems | | | | Inflorescences | | |
| --- | --- | --- | --- | --- | --- | --- | --- | --- | --- | --- | --- |
|  | Vegetative growth (phase 1) | Full flowering (phase 2) | Fruit setting (phase 3) | Mean | Vegetative growth (phase 1) | Full flowering (phase 2) | Fruit setting (phase 3) | Mean | Full flowering (phase 2) | Fruit setting (phase 3) | Mean |
| Prunin | 172.62  ± 26.03 ^a^ | 129.49  ± 10.58 ^b^ | 111.36  ± 34.88 ^b^ | 137.82  ± 36.46 ^B^ | 448.60  ± 61.90 ^ab^ | 420.45  ± 41.02 ^b^ | 532.66  ± 57.11 ^a^ | 467.24  ± 72.09 ^A^ | 75.36  ± 25.19 ^a^ | 18.47  ± 7.05 ^b^ | 46.92  ± 33.93 ^C^ |
| Apigenin-7-O-glucoside | 132.03  ± 15.67 ^a^ | 165.21  ± 48.04 ^a^ | 74.77  ± 20.54 ^b^ | 124.00  ± 48.86 ^A^ | 6.33  ± 2.08 | 8.98  ± 0.83 | 8.36  ± 8.64 | 7.89  ± 5.28 ^B^ | 295.92  ± 79.72 ^a^ | 8.99  ± 3.08 ^b^ | 152.45  ± 154.16 ^A^ |
| Rosmarinic acid | 552.53  ± 194.55 | 518.37  ± 88.03 | 403.25  ± 29.23 | 491.39  ± 139.86 ^A^ | 183.57  ± 95.25 ^b^ | 250.16  ± 52.85 ^ab^ | 327.30  ± 102.40 ^a^ | 253.68  ± 104.40 ^B^ | 224.47  ± 36.14 ^a^ | 27.99  ± 22.37 ^b^ | 126.23  ± 102.73 ^C^ |
| Linarin | 284.38  ± 105.08 ^a^ | 148.26  ± 12.31 ^b^ | 88.13  ± 19.06 ^b^ | 173.59  ± 102.92 ^B^ | 68.77  ± 18.26 ^b^ | 120.13  ± 44.50 ^a^ | 68.29  ± 23.32 ^b^ | 85.73  ± 39.29 ^B^ | 677.34  ± 107.36 ^a^ | 54.29  ± 32.18 ^b^ | 365.82  ± 321.45 ^A^ |
| Didymin | 436.69  ± 103.76 ^a^ | 168.15  ± 21.53 ^b^ | 121.63  ± 12.26 ^b^ | 242.15  ± 151.91 | 232.65  ± 71.08 | 325.50  ± 124.70 | 286.26  ± 145.80 | 281.47  ± 124.10 | 314.09  ± 67.20 ^a^ | 21.61  ± 15.89 ^b^ | 167.85  ± 154.18 |
| Apigenin | 10.87  ± 3.07 ^ab^ | 15.92  ± 5.22 ^a^ | 5.47  ± 0.76 ^b^ | 10.75  ± 5.53 ^B^ | 4.05  ± 1.40 | 3.28  ± 0.66 | 4.08  ± 0.54 | 3.81  ± 1.01 ^C^ | 37.03  ± 8.66 ^a^ | 23.30  ± 10.64 ^b^ | 30.17  ± 11.88 ^A^ |
| The reported values represent the means of three independent replicates in each experimental year (2016, 2017), with standard deviations (± SD) provided. However, the values presented in the last column for each raw material (leaves, stems, and inflorescences) represent the means derived from all replicates across all phenological phases and experimental years for that specific raw material, also accompanied by standard deviations (± SD). The following pairwise comparisons were performed within each row:  1) For a single raw material (leaves, stems, or inflorescences), means from different plant growth phases marked with different lowercase letters (e.g. a, b) differ significantly according to the Tukey test at the significance level α=0.05.  2) For different raw materials (leaves, stems, and inflorescences), means marked with different uppercase letters (e.g. A, B) differ significantly according to the Tukey test at the significance level α=0.05. | | | | | | | | | | | |

**Table S4** Pearson's correlation coefficients between phenolic compound contents (total phenolic content, total flavonoid content and individual phenolic contents) and antioxidant activity (FRAP and DPPH assays)

| **Assay** | FRAP  (Trolox equivalent) | DPPH  (Trolox equivalent) |
| --- | --- | --- |
| Total phenolic content (gallic acid equivalent) | **0.925***** | **0.906***** |
| Total flavonoid content (rutin equivalent) | **0.989***** | **0.991***** |
| Neochlorogenic acid | 0.359 | 0.353 |
| Protocatechuic acid | 0.472** | 0.464** |
| Chlorogenic acid | 0.274 | 0.269 |
| p-Coumaric acid | 0.438* | 0.441* |
| Caffeic acid | 0.382* | 0.391* |
| Luteolin-7-O-glucoside | **0.841***** | **0.838***** |
| Narirutin | 0.305 | 0.312 |
| Prunin | -0.081 | -0.085 |
| Apigenin-7-O-glucoside | 0.541*** | 0.524** |
| Rosmarinic acid | **0.792***** | **0.801***** |
| Linarin | 0.341 | 0.330 |
| Didymin | 0.477** | 0.488** |
| Apigenin | 0.058 | 0.055 |

* Correlation significant at the significance level α=0.01
** Correlation significant at the significance level α=0.001
*** Correlation significant at the significance level α=0.0001

Values in bold denote the strongest correlation

**References**

1. Gontar Ł, Geszprych A, Drutowska A, Osińska E (2024) Phytochemical composition, antioxidant and antimicrobial activity of three *Monarda* species: *M. bradburiana* L. C. Beck, *M. × media* Willd., and *M. punctata* L. Chem Biodivers 21:e202301910. https://doi.org/10.1002/CBDV.202301910

2. Polish Pharmacopoeia (2014) X ed. Office of Registration of Medicinal Products, Medical Devices and Biocidal Products. Polish Pharmaceutical Society, Warsaw

3. Adams RP (2007) Identification of essential oil components by gas chromatography/mass spectrometry, 4th ed. Allured Publishing Corporation, Carol Stream
